# Detached mindfulness as a stand-alone intervention: a Systematic Review and meta-analysis

**DOI:** 10.3389/fpsyt.2026.1771705

**Published:** 2026-04-15

**Authors:** Samuel G. Myers, Stian Solem

**Affiliations:** 1 Centre for Clinical, Cognitive and Affective Sciences, Jerusalem, Israel; 2 Department of Psychology, Norwegian University of Science and Technology, Trondheim, Norway

**Keywords:** detached mindfulness, meta-analysis, metacognition, review, transdiagnostic

## Abstract

**Background:**

Detached Mindfulness (DM) is a central, transdiagnostic technique within Metacognitive Therapy (MCT). It involves increasing meta-awareness of intrusions while decentring from and disengaging with them, and is used to reduce the Cognitive Attentional Syndrome and dysfunctional metacognitive beliefs—key components of the metacognitive model of psychological disorders. Although DM is typically delivered within a full MCT protocol, recent research has begun to evaluate DM as a stand-alone intervention. The current study aimed to systematically review and meta-analyse its effects.

**Methods:**

Studies were included in the systematic review if they examined DM delivered as a stand-alone intervention in clinical or non-clinical samples. Searches were carried out in PubMed, Scopus, Web of Science, and Google Scholar in May 2025. Methodological quality and risk of bias were assessed using an adapted quality appraisal checklist. Random-effects meta-analyses were conducted for clinical trials, and narrative synthesis for experimental studies.

**Results:**

Fourteen studies met inclusion criteria, representing twelve independent samples exposed to DM (*N* = 256; all aged ≥17). Three samples evaluated DM as a stand-alone treatment in clinical trials (two in obsessive–compulsive disorder and one in panic disorder). Of the remaining nine samples, seven (one clinical, six non-clinical) examined the effects of DM on experimentally induced psychological symptoms, and two (non-clinical) on pre-existing symptoms. Across the three clinical trials, DM was associated with large symptom reductions (pooled Hedges’ *g* for primary outcomes = −1.80, 95% CI [−2.84, −0.76]; pooled *g* for depressive symptoms measured as a secondary outcome = −1.15, 95% CI [−2.23, −0.08]. Of the remaining nine samples, eight reported beneficial effects of DM on at least one outcome, with significant effects typically in the medium-to-large range.

**Conclusions:**

These findings provide converging, but still limited, evidence that DM as a stand-alone intervention is associated with improvements in psychological symptoms and related processes. The results have implications for future component analyses of MCT and for the development of brief, transdiagnostic metacognitive interventions. However, there are significant limitations to the current research base including the small number of studies, methodological shortcomings, and lack of long-term follow-up; these are discussed as well as suggestions for future studies.

## Introduction

Metacognitive Therapy (MCT; 1) is a transdiagnostic psychological treatment grounded in the Self-Regulatory Executive Function (S-REF) model (2). According to this model, emotional disorders are maintained by a particular way of responding to internal experiences—such as thoughts, feelings, and memories—known as the Cognitive Attentional Syndrome (CAS). The CAS consists of perseverative thinking styles, particularly worry and rumination, attentional fixation on threat, and unhelpful coping behaviours that prolong distress. The CAS is driven by dysfunctional metacognitive beliefs (e.g., beliefs about the need to worry or the danger of thoughts). MCT aims to modify these beliefs and remove the CAS, thereby restoring adaptive self-regulation. Two meta-analyses suggest that MCT is an effective treatment for emotional disorders and may be superior to other psychotherapies (3, 4).

MCT consists of several therapeutic components and techniques. A central intervention is Detached Mindfulness (DM), which represents the opposite mode of processing to the CAS. In DM, individuals notice an intrusion, adopt a decentred stance toward it, refrain from further conceptual processing, and allow the intrusion to occupy its own mental space without responding to it. Thoughts are experienced as transient mental events, that do not require evaluation, control, or action.

According to 1, (5), DM comprises six essential elements: 1) *Meta-awareness* – noticing thoughts and mental activity as events in the mind; 2) *Cognitive decentring* – recognising that thoughts are not facts and do not define the self; 3) *Flexible attention* – the ability to shift attention freely rather than becoming fixed on the intrusion; 4) *Low conceptual processing* – suspending analysis, elaboration, and meaning-making in response to the intrusion; 5) *Low goal-directed coping* – refraining from attempts to control, neutralise, suppress, or otherwise cope with the intrusion; and 6) *Self-awareness as observer –* a state of awareness in which the person experiences a sense of self as an observer of mental events but distinct from them.

As can be inferred from these six elements, DM is used in MCT not only to reduce the CAS but also to modify metacognitive beliefs (e.g., beliefs about the uncontrollability of thoughts), and to help patients adopt a metacognitive mode in which thoughts and feelings are experienced as mental phenomena rather than objective reality. It is also used to reduce the influence of cognitive content on the individual’s sense of self.

DM can be differentiated from many mindfulness-based interventions (see 1, 5 for a full discussion). DM does not involve increasing awareness of sensory experiences or the present moment. It also does not involve attentional anchoring or the use of meditation or breathing exercises. These techniques can require sustained attentional focus on internal experiences which is incompatible with DM. Instead, DM focuses specifically on meta-awareness of thoughts, in a decentred manner, without further processing, in a way that is incompatible with the CAS. This also differentiates DM from some forms of acceptance-based approaches where imagery is used to transform thoughts. However, some metacognitive constructs specified in DM may overlap with mindfulness-based constructs (e.g., non-judging of inner experience), while others (e.g., observing and describing) are more unique to mindfulness-based approaches (6).

1, (5) described ten different methods to facilitate DM. These include exercises such as the tiger task, in which the individual imagines a tiger and observes what happens without intervening; the free-association task, where a list of words—first neutral and then including triggering words—is read aloud while the individual simply listens without actively generating or preventing images; and the suppression versus counter-suppression experiment, where DM is contrasted with thought suppression. The ten methods also include the use of metaphors, such as the train metaphor—in which thoughts are compared to trains arriving and leaving a station—and the cloud metaphor, where thoughts are compared to clouds that float by and cannot be influenced, making attempts to control or change them unnecessary.

An additional technique in MCT that is separate from but related to DM is the Attention Training Technique (ATT). ATT is a 15-minute auditory exercise with three components: selective attention, attention switching, and divided attention. It was designed to increase attentional flexibility and reduce perseverative conceptual activity. Although ATT—like DM—was originally developed as one component within the wider MCT protocol, several studies have investigated ATT as a stand-alone intervention, and two systematic reviews support its effectiveness (7, 8), reporting large effect sizes for anxiety and depression (8). Furthermore, evidence suggests that improvements in attentional control may be a candidate mechanism of change in ATT (7).

The findings from ATT suggest that individual techniques within MCT may be effective when delivered on their own, which has encouraged growing interest in evaluating DM as a stand-alone intervention. Examining single components of MCT is important both for understanding the therapeutic mechanisms of the full treatment and for developing brief, transdiagnostic metacognitive interventions to alleviate distress. The present paper therefore aimed to conduct, to our knowledge, the first systematic review and meta-analysis of DM delivered as a stand-alone intervention. The key question addressed in this initial study was whether DM, when given alone, produces beneficial effects and, if so, how strong these effects are.

## Methods

### Review design

The review was carried out based on PRISMA guidelines. Although a protocol was not pre-registered, the review was guided by decisions made in advance regarding eligibility criteria, search strategy, data extraction, the use of meta-analysis where studies were sufficiently homogeneous and using random-effects models to address the anticipated levels of heterogeneity. Other aspects of the review, such as analysing potential causes of heterogeneity, were developed during the review process.

### Eligibility criteria

Studies were included if they met the following criteria:

1. Published in peer-reviewed journals and written in English.
2. Included human participants, clinical or non-clinical, of any age.
3. Employed DM, as defined in MCT, as a stand-alone intervention.
4. Reported at least one psychological outcome (e.g., frequency of intrusions).
5. Used clinical trials or experimental designs, including randomised controlled trials (RCTs), open trials, and both randomised and non-randomised experimental studies.

Studies were excluded if DM was combined with other interventions, including medication or other metacognitive techniques. Studies were included if only psychoeducation was provided alongside DM, or if participants were on pre-existing medication, not part of the intervention.

DM interventions were defined as those that: 1) explicitly described the intervention as DM and met the core criteria of meta-awareness with decentring and without further processing, or 2) employed one of the techniques described by 1, (5) as ways of inducing DM.

One such technique, the *Clouds Image*, involves the following instruction (5; p349):

“One way to help you achieve detached mindfulness is to think of your thoughts as clouds floating in the sky. It would be unnecessary and impossible to push clouds away and control their movements. Treat your thoughts and feelings as clouds. Imagine your thoughts printed on them and allow them to occupy their own space as they pass you by”.

However, (5) explicitly noted that this imagery-based procedure involves a degree of active engagement with thoughts and therefore does not represent a pure form of DM, a position that was further clarified in later developments of DM (1). Although this suggests that the technique should not be considered a core DM procedure, studies employing this method were included under a broader operational definition of DM, as the technique was discussed in the earlier account and as it may facilitate a detached stance toward thoughts, particularly as the technique incorporates a “thoughts as clouds” metaphor. Such studies were classified as reflecting partial fidelity to DM and were interpreted accordingly. Where an intervention was described as DM but included techniques not described by 1, (5), with insufficient reporting to allow judgement of fidelity, attempts were made to contact the study authors for clarification; where uncertainty remained, such studies were included with explicit caveats in interpretation.

To maximise comprehensiveness in this initial review no minimum criteria were set for the duration of the DM intervention or the format of instruction used.

Studies meeting eligibility criteria were grouped according to study design for synthesis. Clinical trials evaluating DM as a stand-alone treatment in clinical populations were synthesised quantitatively using meta-analysis, whereas experimental studies were synthesised narratively due to heterogeneity in design and outcomes.

### Search strategy

In May 2025, a search was made of PubMed, Web of Science, and Scopus using the exact phrase “Detached Mindfulness”. Searches in Web of Science and Scopus were also restricted to studies published in English.

The following search strings were used.

PubMed: “Detached Mindfulness” [Title/Abstract].

No date, language or other filters were applied.

Scopus: TITLE-ABS (“Detached Mindfulness”).

This was limited to English-language publications but no other limitations were applied.

Web of Science: TS (“Detached Mindfulness”).

This was limited to English-language publications but no other limitations were applied.

Additionally, the first 200 results for the same phrase in Google Scholar were examined.

The search string for Google Scholar was “Detached Mindfulness”.

All search results were exported to Rayyan for duplicate removal and screening.

Grey literature sources (e.g., conference abstracts, posters, dissertations, and preprints) and clinical trial registries were not systematically searched as the review focused on peer-reviewed empirical studies to ensure methodological quality and allow study appraisal. The review was restricted to English-language publications to ensure that both authors could assess and extract data from all included studies in a language they fully understand.

### Selection process

Titles and abstracts were independently screened by both authors using the eligibility criteria, with discrepancies resolved through discussion. Full-text articles of potentially eligible studies were subsequently assessed using the same criteria, and disagreements were similarly resolved through discussion.

### Data abstraction

The following information was extracted from all articles by each author independently, where available:

1. Study details, including year of publication, country where the study was conducted, and journal of publication.
2. Study design, including trial or experimental type.
3. Sample characteristics, including sample size, control group, recruitment method, population, eligibility criteria, age, and gender.
4. Intervention details, including how DM and any control interventions were delivered, number of sessions, and session length.
5. Outcome measures, including primary and secondary outcomes, and dropouts.
6. Results, including within- and between-group changes on outcome measures.

### Quality assessment

The methodological quality of the included studies was assessed using an adapted checklist drawn from the National Institutes of Health’s Quality Assessment Tool for Before–After (Pre–Post) Studies With No Control Group (9). The tool was modified to accommodate both experimental and intervention designs and to evaluate fidelity to Metacognitive Theory as well as reporting transparency.

A customised tool was used as the review included heterogeneous study designs (i.e. randomised controlled trials, open trials, and experimental studies). Design-specific tools such as Cochrane RoB 2 (optimised for randomised controlled trials; 10), ROBINS-I (developed for non-randomised studies of interventions; 11), and JBI critical appraisal tools (tailored to specific study designs; 12) were therefore not used, as their design and scoring frameworks are less suitable for synthesising evidence across diverse empirical approaches.

The adapted checklist comprised ten domains assessing: 1) Clarity of research objectives, 2) Adequacy of population description, 3) Sample size justification, 4) Clarity of intervention description and replicability, 5) Theoretical fidelity of the DM intervention, 6) Outcome measures, 7) Timing of measurements, 8) Data completeness, 9) Appropriateness and adequacy of statistical methods, and 10) Reporting transparency.

Each domain was rated on a 3-point scale, scoring 1 if the criterion was met, 0.5 if it was partially met or unclear, and 0 if it was not met. Total scores reflected overall methodological quality. The two reviewers independently rated all studies, and discrepancies were resolved through discussion.

### Synthesis strategy

Form of synthesis was decided based on homogeneity of design, population, and outcome measures. The clinical trials were judged to have sufficient homogeneity to warrant quantitative synthesis using meta-analysis, whereas for the experimental studies a narrative synthesis was used as designs and outcomes were heterogeneous.

### Meta-analyses

For the clinical trials, meta-analyses were conducted on both primary and secondary outcomes. All analyses were performed using R (version 4.5.2) with the *metafor* package (version 4.8.0; 13). Standardized mean change (Hedges’ *g*) values were calculated for pre–post differences within each study, using an assumed pre–post correlation of *r* = 0.5. Random-effects models with restricted maximum likelihood (REML) estimation were applied to account for between-study variability. Heterogeneity was evaluated using *Q*, *I*², and τ² statistics. Owing to the small number of studies, formal statistical analyses of heterogeneity (e.g. subgroup analysis) were not used, instead study differences were examined descriptively. Sensitivity analyses were performed across alternative assumed pre–post correlations (*r* = 0.3, 0.5, 0.7) to assess robustness. Forest plots were used to show individual and pooled effect sizes visually.

As fewer than ten studies were included in each analysis, funnel plot inspection and formal tests for publication bias (e.g., Egger’s regression) were not carried out, consistent with Cochrane recommendations that such methods are unreliable in small meta-analyses.

### Other analyses

In studies not included in the meta-analyses, effect sizes were extracted when reported, otherwise, where possible, standardised effect sizes (*g*, *r* or phi) were calculated from significant outcomes using R with the *metafor* or *esc* packages (14) to provide a standardised measure of effect size across studies. Effect sizes were interpreted using conventional guidelines: for *g*, small (*g* = 0.20), medium (*g* = 0.50), and large (*g* ≥ 0.80) for *r* and phi, 0.10 small, 0.30 medium and ≥ 0.50 large.

### Risk of bias due to missing results

For the clinical meta-analyses, assessment of reporting bias using funnel plots or statistical tests was not conducted due to the small number of studies, consistent with Cochrane recommendations. For the experimental studies, which were synthesised narratively, statistical assessment of reporting bias was not applicable. Although comprehensive searches were conducted, the inclusion of published studies only, means that reporting bias cannot be ruled out.

### Certainty of evidence

Certainty of evidence was evaluated based on the adapted Quality Checklist, number of studies, sample sizes, study design and consistency of findings.

## Results

### Search results

**Figure 1:**
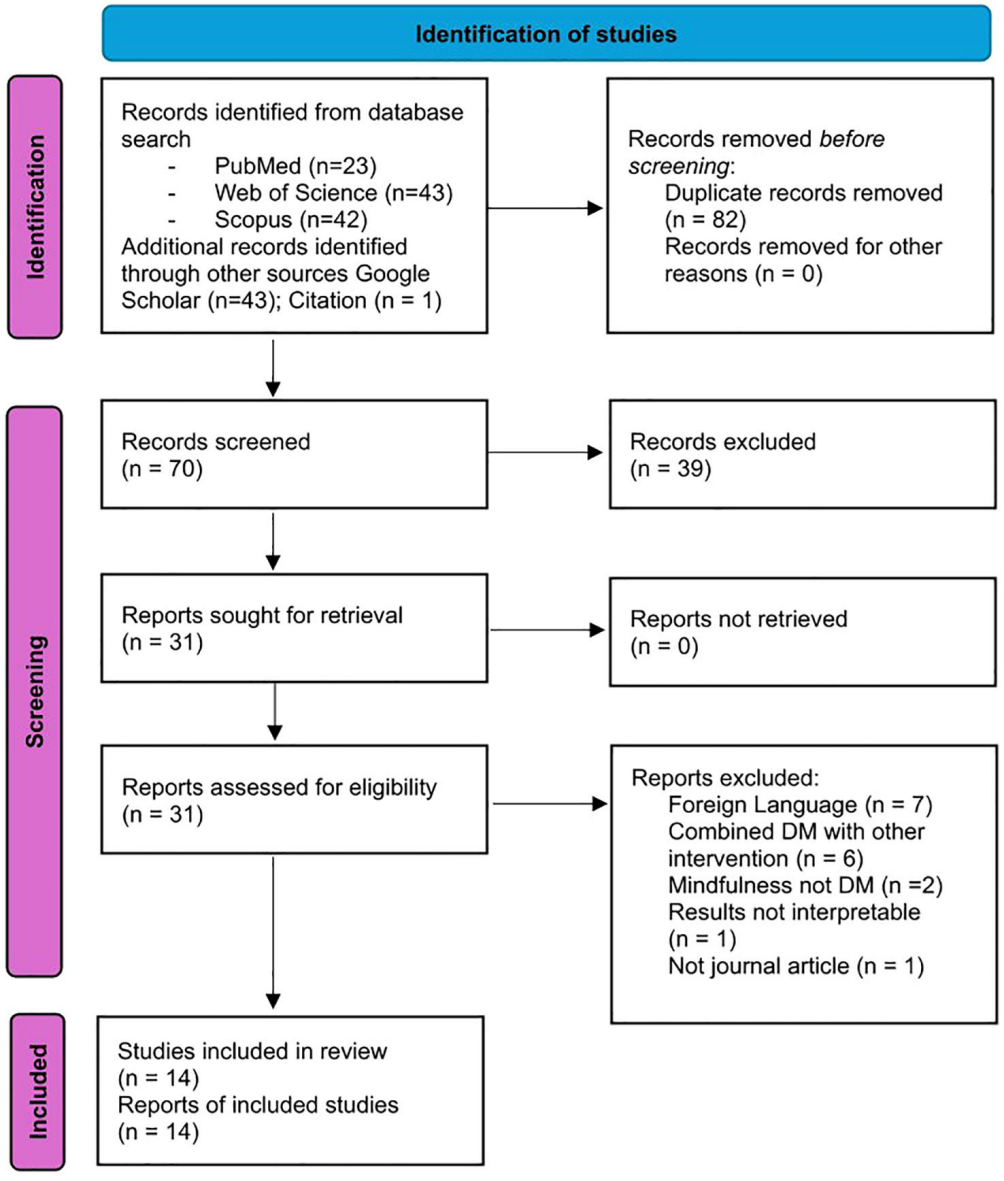
Flow diagram of the study selection.

A PRISMA diagram showing the search results and selection process is shown in **Figure 1**.

As shown, the searches identified 152 articles, of which 82 duplicates were removed using Rayyan. The remaining 70 records were screened, and 39 were excluded after examination of the title and/or abstract, as they clearly did not meet the eligibility criteria for inclusion. The remaining 31 articles were screened using the full text. Of these, 17 were excluded for the following reasons: 1) The article was not written in English; 2) DM was combined with other interventions (e.g., challenging metacognitive beliefs or medication); 3) The study employed mindfulness rather than DM; 4) The results were not interpretable, as the authors appeared to have combined their experimental and control groups; 5) The article was not published in a peer-reviewed journal. **Table 1** presents the individual studies excluded at the full-text stage along with reason of exclusion.

**Table 1:** Studies excluded at full-text screening and reasons for exclusion.

Study	Reason for exclusion
Abbas et al. (15)	Not written in English
Ahmadpanah et al. (16)	Included ATT (Attention Training Technique)
Almosawy et al. (17)	Combined DM with medication
Asgarabad et al. (18)	Combined DM with poetry and ATT
Bakhtiari et al. (19)	Not written in English
Bakhtiari et al. (20)	Not written in English
Firoozabadi et al. (21)	Not written in English
Ghasem et al. (22)	Not written in English
Gholami et al. (23)	Not written in English
Listiyandini et al. (24)	Used mindfulness rather than DM
Mikaeili et al. (25)	Combined DM with ATT
Modini and Abbott (26)	Combined DM with challenging positive metacognitive beliefs
Nuske et al. (27)	DM instruction included mindfulness elements inconsistent with Metacognitive Theory namely raising awareness of bodily sensations and a greater focus on raising awareness of thoughts and feelings than in standard DM
Pour et al. (28)	Not written in English
Qamar et al. (29)	Not interpretable — combined results for experimental and control groups
Raffone et al. (30)	Poster presentation (not peer-reviewed)
Wahl et al. (31)	Used mindfulness rather than DM

DM, Detached Mindfulness; ATT, Attention Training Technique. Studies are listed alphabetically by first author’s surname. Full bibliographic references for these excluded studies are provided in the reference list.

A total of 14 articles met the inclusion criteria. Summary descriptive information of included studies is shown in **Table 2**.

**Table 2:** Summary descriptive information of reviewed DM-studies.

Study, country, and design	Sample	Conditions and length	Measures	Outcomes and effect sizes**
Akdağ et al. (32) Netherlands RCT	120 undergraduate students. 92.5% women. Mean age: 19.65, range: 18–24.	DM, *n* = 30. Video using four DM techniques from (5). ATT, *n* = 30. Slow Breathing, *n* = 30. Active control, *n* = 30. 12 minutes.	STAI State metacognition HRV	Significant increase in HRV from pre to post in DM group vs. control but not in metacognition or anxiety.
Atmaca et al. (33)* Turkey Open trial	17 patients with OCD. 61.1% women. Mean age: 34.29 (7.55).	Psychoeducation then DM using 1) word association task first part, 2) Tiger task, 3) DM to intrusive thoughts. DM homework. 4 weekly sessions. Minutes: na.	Y-BOCS BDI BAI	Significant improvements in symptoms of OCD, depression, and anxiety. Y-BOCS, *g* = -2.25 BDI, *g* = 1.73 BAI, *g* = -2.59
Atmaca et al. (34)* Turkey Open trial	11 patients with panic disorder. 63.6% women. Mean age: 36.63, (8.15).	Psychoeducation then as above plus 1 and 3 with taking a few steps back instruction. DM homework. 4 weekly sessions. Minutes: na.	PDSS BAI BDI	Significant improvements in symptoms of panic, depression, and anxiety. PDSS, *g* = -2.53 BDI, *g* = -1.73 BAI*, g* = -1.91
Bolzenkötter et al. (35) Germany RCT	100 subjects with elevated repetitive negative thinking (RNT). 72.0% women. Mean age: 33.8, (11.2).	DM, *n* = 50. Imagine thoughts as clouds in sky, leaves on tree or trains in a station. Active control, *n* = 50. Same audio as DM but no reference to participants’ thoughts. 4.5 minutes 3 times a day for 5 days	EMA: RNT, negative affect, and positive affect.	Significant improvements in RNT, negative and positive affect in both conditions.
Caselli et al. (36) Italy Counterbalanced repeated-measures design	8 patients with alcohol use disorder. 50.0% women. Mean age: 42.0 (4.1), range: 35–50.	DM and brief exposure DM: Step back and not respond to thoughts. Telephone metaphor. Control: Habituation rationale 1 session. Minutes: na.	VAS scales: distress, urge to drink, fear of alcohol-related thoughts, metacognitive beliefs.	Significantly greater decreases in meta-appraisal, metacognitive beliefs, distress, and urge to use alcohol for DM. *r =* –0.67 to –0.88
Chittaro and Vianello (37) Italy Counterbalanced within-subjects design	Students; naive meditators (*n* = 22). Women: na. Mean age: 23.95 (2.15), range: 19-28.	DM = Imagine thoughts written on clouds, allow them to occupy their own space as they pass by. AEON: app where triggers are written on cards and waves can be used to blur the text. CARD: Toss cards with intrusion written into a wastepaper basket. 1 session. Minutes: na.	Decentring from TMS Pleasure Difficulty	No difference on decentring between DM and AEON. DM significantly less pleasant than AEON and more difficult than both AEON and CARD.
Gkika and Wells (38) England Cross-over repeated measures design	12 subjects with high social anxiety symptoms. 100% women. Mean age: 19.17 (1.69).	DM: Suppression/counter-suppression experiment and word association task. Thought evaluation (TE): Socratic questioning. 20 minutes.	S-ASBQ Observer perspective State anxiety Belief in negative thoughts	DM led to significant reductions in anticipatory processing, observer perspective, negative beliefs and anxiety. These were significantly greater for all measures, apart from anxiety, than TE. *r* = -63 to -67
Gold and Smout (39) Australia Pre-post design	23 community participants. 81.8% women. Mean age: 28.50 (11.16).	DM: “Have you tried to stop criticising yourself? Distinction between intrusion and rumination, thought suppression, tiger task, DM to triggers. Homework DM. 50 minutes.	SCRS FSCRS-HS and -IS MSCQR-N and -P DASS-A and -D RSES EISS-E and -I, MpoD-t-M, -D and -N Daily diary ratings	Significant decreases in self-critical rumination, metacognitive beliefs and anxiety *g* = .35 to 1.10 Also significant increases on two measures of decentring processes *g* = -1.11 and -1.32
Hansmeier et al. (40) Germany RCT	57 nonclinical with OCD symptoms. 94.3% women. Mean age: 26.5, (12.0).	Dm, *n* = 27. Cloud Metaphor (1) and Socratic questioning Control, *n* = 30, info on mnemonics. 1 session. Minutes: na.	TAF SGS	DM failed to show a preventive effect on TAF and shame.
Ludvik and Boschen (41) Australia RCT	65 undergraduates. 73.8% women Mean age: 22.72 (7.23), range: 17-53.	DM, *n* = 21. Be aware of thoughts, but not get caught up in believing or evaluating them. Cognitive restructuring (CR), *n* = 22. Control, *n* = 22. Read astronomy article. 1 session. Minutes: na.	CTQ	DM and CR more effective in reducing rechecking than control. *g* = –0.38 to –0.52 DM also ameliorated effects of reduced memory distrust.
Rupp, Jürgens et al. (42)* Germany RCT	64 patients with OCD. 59.1% women. Mean age: 30.81 (9.48).	DM, *n* = 21. Different examples and metaphors, guided imagery, involving visualisation of obsessions and a shift to detached, passive observation. CR, *n* = 22. Waitlist, *n* = 21. 4 sessions of 5–10 minutes.	Y-BOCS BDI-II	Both CR and DM led to significant reductions in symptoms which were superior to waitlist. No difference between CR and DM. Y-BOCS, *g* = -0.91 BDI-II, *g* = -0.16
Rupp et al. (43) Germany RCT	As above.	As above.	EMA: symptoms and use of DM and CR.	DM and CR behaviours increased from pre to post (except “*Come and Go”)*
Rupp, Falke et al. (44) Germany RCT	As above.	As above.	EMA: symptoms, avoidance, obsessions.	Reduction of symptoms, avoidance, and obsession. No group differences.
Wells and Roussis (45) England RCT	56 students. 57.1% women. Mean age: 21.5, range: 18–42.	DM, *n* = 14. “Acknowledge the presence of it [image] in your mind and let it occupy its own mental space and possess its own behaviour. Do not interfere with the intrusion or try to control it”. Acceptance, *n* = 14. Imaginal exposure, *n* = 14. Control group, *n* = 14. 5 minutes.	Frequency of intrusive images.	The DM group had significantly lower frequency of intrusions compared to control *g* = 0.95***

ATT, attention training technique; BDI, Beck Depression Inventory (46); BAI, Beck Anxiety Inventory (47); CTQ, Checking task and questionnaire (48); DASS-A, Depression Anxiety Stress Scale Anxiety subscale; (DASS)-D Depression subscale, (49); EISS-E, External Internal Shame Scale External subscale; (EISS)-I, Internal subscale (50); EMA, ecological momentary assessment; FSCRS-HS, Forms of Self Criticism and Reassurance Scale-Hated Self subscale; (FSCRS)-IS, -Inadequate Self subscale (51); HRV, Heart Rate Variability; OCD, Obsessive-Compulsive Disorder; MpoD-t-M, Metacognitive Processes of Decentring scale Trait version Meta-awareness subscale; (MpoD-t)-D, Disidentification with internal experience subscale; (MpoD-t)-N, Nonreactivity subscale (52); MSCQR-N, Metacognitions about Self-Critical Rumination Negative subscale; (MSCQR)-P, Positive subscale (53); PSWQ, Penn State Worry Questionnaire (54); PDSS, Panic Disorder Severity Scale (55); RSES Rosenberg Self-Esteem Scale (56); S-ASBQ, State-Anticipatory Social Behaviours Questionnaire (57); SCRS, Self-Critical Rumination Scale (58); SGS, Shame and Guilt Scale (59); STAI, State-Trait Anxiety Inventory (60); TAF, Thought Action Fusion scale (61); TCQ, The Thought Control Questionnaire (62); TMS, Toronto Mindfulness Scale (63); TQ-E, the extended version of the Thoughts Questionnaire (64); Y-BOCS, Yale-Brown Obsessive Compulsive Scale (65). *Studies included in meta-analysis. **Effect sizes shown are for the DM intervention and are within-group pre–post changes unless otherwise indicated. Where effect sizes were not reported, they were calculated from reported statistics where possible. For Rupp, Jürgens et al., the reported effect size was recalculated to ensure consistency with the analytic approach used across studies. ***In Wells & Roussis, only the between-group effect size relative to a non-active control condition was reported and this is included in the table.

### Characteristics of the included samples and study types

Fourteen articles were included in the review, corresponding to twelve distinct participant samples, as three articles (42–44) presented different outcomes derived from the same underlying study population. Across these twelve samples, the number of participants receiving DM (excluding control conditions) ranged from 8 to 50, with a total of 256 participants. All participants were aged 17 or over.

Of the twelve samples, three were clinical trials (two open trials and one randomised controlled trial), two of which treated patients with obsessive–compulsive disorder (OCD) (33; *n* = 17; 42; *n* = 20 in the DM condition), and one treated patients with panic disorder (33; *n* = 11).

Of the remaining nine samples, seven experimentally induced symptoms or triggers to examine the immediate effects of DM on specific outcome measures. One induction study involved a clinical sample of patients with alcohol use disorder (36; *n* = 8), while the remaining six used non-clinical samples, with DM sample sizes ranging from 12 to 30 participants.

The final two samples examined the effects of DM on pre-existing symptoms rather than experimentally induced states, specifically self-critical rumination (39; *n* = 23) and negative repetitive thinking (35; *n* = 50 in the DM condition), in non-clinical populations.

### Quality assessment

Scores on the quality checklist across the 14 included studies ranged from 6.0 to 9.0 on a 0–10 scale, with higher scores indicating better methodological quality. Most studies demonstrated clear research objectives, adequate descriptions of intervention procedures, good theoretical fidelity to DM principles, and appropriately timed pre- and post-intervention assessments.

All studies provided basic descriptions of their participant samples; however, clinical characteristics were often absent or only partially reported, particularly in experimental studies. Although outcome measures generally had good face validity, several studies included measures that had not been validated. With one exception, studies employed appropriate statistical analyses; however, reporting of key statistical assumptions (e.g., normality, presence of outliers, or handling of non-normal data) was frequently absent.

Most studies did not report a sample size justification or power analysis. In addition, incomplete reporting of missing data and attrition was common, resulting in reduced scores for data completeness. Although outcomes were generally reported transparently, two studies were only given a. 5 score on this item for not providing pre- and post-intervention descriptive statistics that would have allowed calculation of standardised effect sizes.

A summary of the quality assessments is presented in **Table 3**.

**Table 3:** Quality assessment of included studies.

Study	D1	D2	D3	D4	D5	D6	D7	D8	D9	D10	Sum
Akdağ et al. (32)	1	1	.5	1	1	.5	1	1	1	.5	8.5
Atmaca et al. (33)	1	.5	0	1	1	1	.5	.5	.5	1	7.0
Atmaca et al. (34)	.5	1	.5	.5	1	1	.5	.5	0	1	6.5
Bolzenkötter et al. (35)	1	.5	0	1	1	.5	1	.5	.5	1	7.0
Caselli et al. (36)	1	1	0	1	1	.5	1	.5	.5	.5	7.0
Chittaro and Vianello (37)	1	.5	0	1	.5	.5	.5	.5	1	1	6.5
Gkika and Wells (38)	1	.5	.5	1	1	.5	1	.5	.5	1	7.5
Gold and Smout (39)	.5	1	0	1	1	.5	1	.5	.5	1	7.0
Hansmeier et al. (40)	1	1	1	1	1	.5	1	1	.5	1	9.0
Ludvik and Boschen (41)	1	.5	0	.5	.5	.5	1	.5	.5	1	6.0
Rupp, Jurgens et al. (42)	1	1	.5	.5	1	1	1	1	.5	1	8.5
Rupp et al. (42)	1	1	0	.5	1	.5	1	1	1	1	8.0
Rupp, Falke et al. (44)	1	1	0	.5	1	.5	1	1	1	1	8.0
Wells and Roussis (45)	1	.5	0	1	1	.5	.5	0	.5	1	6.0

D1, Clarity of research objectives; D2, Adequacy of population description; D3, Sample size justification; D4, Clarity of intervention description and replicability; D5, Theoretical fidelity of the DM intervention; D6, Outcome measures; D7, Timing of measurements; D8, Data completeness (clarity of reporting of missing data and dropouts; D9, Appropriateness and adequacy of statistical methods; and D10, Reporting transparency. Scores: 1= criterion met, 5, criterion partially met or unclear, 0, criterion not met.

### DM intervention procedures across studies

All studies grounded their procedures in the metacognitive theory of DM or in techniques specifically outlined by Wells 1, (5) for inducing DM. However, the format and length of the DM intervention varied across studies. In the three clinical trials, DM was delivered across four sessions and embedded within a metacognitive understanding of the particular disorder. These trials began with psychoeducation framed from this perspective, followed by several structured DM techniques, guided practice applied to disorder-specific intrusive thoughts, and homework assignments to consolidate DM skills. The other studies used briefer DM interventions. The seven studies that induced symptoms used single-session DM interventions, whereas the two studies examining pre-existing symptoms provided an initial DM instruction or session followed by several days of practice—either for one week whenever they experienced a trigger (39) or for five days, three times per day (35).

Across studies, DM was induced in a range of different ways. One study (45) p 542 used only a single spoken instruction (“acknowledge the presence of it in your mind and let it occupy its own mental space and possess its own behaviour. Do not interfere with the intrusion or try to control it.”). In contrast, most studies employed at least one metaphor or a technique outlined by Wells (5). Three techniques specified in more than one study were the Tiger Task, the Word Association Task, and the Suppression/Counter-suppression exercise.

Chittaro and Vianello (37) used a technique for inducing DM described by Wells (5), in which individuals imagine their thoughts as written on clouds, watching them move by without engagement. As discussed earlier, Wells 1, (5) notes that this version still involves a degree of active engagement with thoughts so does not represent pure DM. Although, as explained earlier, use of this technique was included in the review, this intervention was marked in the Quality Checklist as having only partial fidelity to how DM is construed in Metacognitive Theory and the results were examined accordingly. Wells 1, (5) explained that cloud imagery can be used as a metaphor for pure DM. This was the approach used by Hansmeier et al. (40), who asked participants to imagine thoughts as clouds passing across the sky, part of the atmosphere’s natural self-regulation and not something they could influence. This intervention was therefore rated as having full fidelity to DM as conceptualised in Metacogntive Theory.

Bolzenkötter et al. (35) instructed participants to use imagery metaphors with thoughts represented as clouds in the sky, leaves on a river, or trains at a station. Participants were told to watch the ebb and flow of their thoughts without engaging with them. Bolzenkotter et al. reported that the audio transcript used in their study was adapted from a mindfulness exercise (“leaves on a river”; 66) and that the same script was also applied to two DM metaphors—clouds in the sky and trains at a station. Although they stated that key characteristics of DM were incorporated in the exercise, the article did not provide sufficient detail as to how the original mindfulness exercise was adapted. We contacted the author to request clarification, but no response was received at the time this article was submitted. Accordingly, the degree of adherence to DM principles remains uncertain, particularly as the original exercise came from a different therapeutic modality. The study was nonetheless included because the article stated that the procedure was faithful to key aspects of DM, but this uncertainty in DM fidelity is taken into account when interpreting its results.

In most studies the DM intervention was delivered verbally-in person or by audio recording, one used an animated video, and one provided written instructions only. Some studies required participants to practice the technique after instruction, whereas others presented the instruction or metaphor without guided practice.

Only four studies assessed either the difficulty of DM or how well participants were able to implement it. Akdağ et al. (32) delivered DM digitally using two videos (instructional and practice) and stated that they drew on four DM techniques described by Wells. They reported that participants rated DM as significantly more difficult than the two comparison techniques (ATT and slow breathing). Bolzenkötter et al. (35) asked participants to practice DM over several days using audio-based exercises and to rate how well they were able to implement the technique; average ratings indicated moderate-to-good implementation ability. Rupp et al. (43), using Ecological Momentary Assessment (EMA), assessed the perceived difficulty of applying DM in daily life. On a 0–6 Likert scale (0 = not at all; 6 = very much), the mean difficulty rating for DM was 2.68, indicating low-to-moderate difficulty; the corresponding rating for cognitive restructuring did not differ significantly. Finally, Chittaro and Vianello (37) compared a cloud-imagery task (see earlier discussion for limitations of this method) with two non-DM techniques and found that the cloud task was rated as more difficult than both comparison conditions.

### Outcomes

#### Clinical trials

The two open trials and the RCT were judged to be sufficiently homogeneous in design, implementation of DM, and outcome measures to permit meta-analysis. The results of these analyses are presented below. Given that only three studies were available, neither visual inspection of funnel plots nor formal tests of publication bias were conducted, as such approaches are not informative in very small meta-analyses.

**Figure 2:**
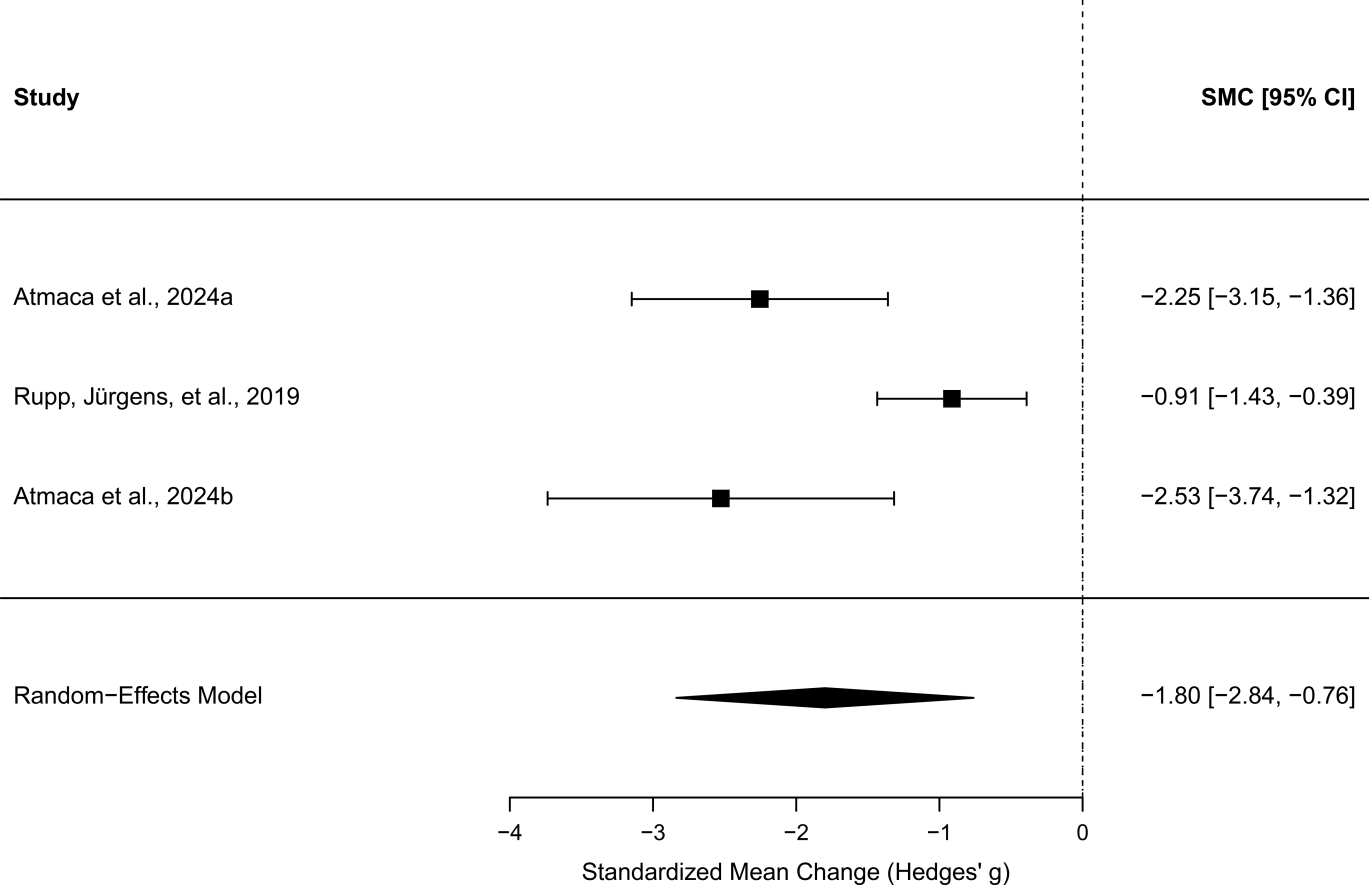
Forest plot showing standardised mean change (Hedges' g) in primary symptom outcomes following Detached Mindfulness across clinical trials. Negative values indicate symptom reduction. Square size reflects study weighting, horizontal lines represent 95% confidence intervals, and the diamond indicates the pooled random-effects estimate.

All three studies reported pre–post data on primary outcomes (the Y-BOCS in the two OCD studies and the PDSS in the Panic study). A random-effects meta-analysis (k = 3 studies, total N = 48) indicated a large overall pre–post reduction in the primary outcome measures following DM, *g* = −1.80 [95% CI −2.84, −0.76], *z* = −3.40, *p* = .001. Heterogeneity was substantial (*Q*(2) = 10.18, *p* = .006; *I*² = 77.7%, τ² = 0.64)), reflecting variation in effect magnitude across studies. Sensitivity analyses varying the assumed pre–post correlation (*r* = 0.3, 0.5, 0.7) produced comparable results (*g* = -1.55 to -2.24), indicating that the overall effect was robust to this assumption. A forest plot showing standardised mean change (Hedges’ *g*) for pre–post reductions in primary symptoms across studies is shown in **Figure 2**.

Please note that effect sizes reported in Rupp, Jürgens, et al. (42) differ from those presented here due to differences in calculation methods. Rupp, Jürgens, et al. computed Cohen’s d using a fixed baseline standard deviation and pooled standard deviations across the DM and Cognitive Restructuring groups, and did not incorporate pre–post correlations. In contrast, to ensure consistency across studies and in line with standard meta-analytic practice, the present review calculated standardised mean change effect sizes (*g*), using both pre- and post-intervention standard deviations and an assumed pre–post correlation of *r* = .50.

As shown, Atmaca et al. (33) and Atmaca et al. (34) had similar very large effect sizes, while Rupp et al. (42) had a smaller but still large effect size.

#### Secondary outcomes

**Figure 3:**
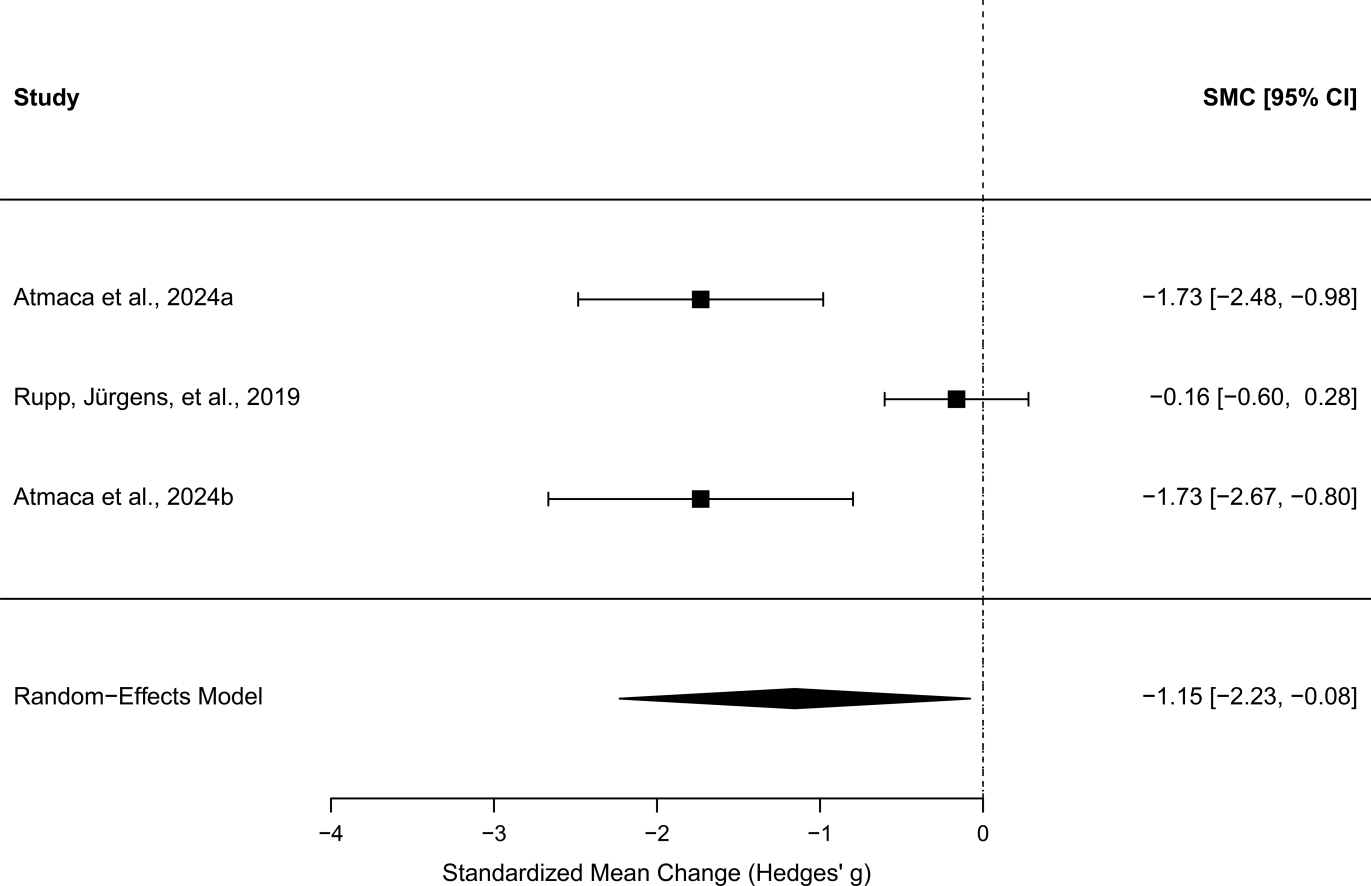
Forest plot showing standardised mean change (Hedges' g) in secondary (depression) symptom outcomes following detached mindfulness across clinical trials. Negative values indicate symptom reduction. Square size reflects study weighting, horizontal lines represent 95% confidence intervals, and the diamond indicates the pooled random-effects estimate.

The three studies also reported pre–post Beck Depression Inventory (BDI) scores. A random-effects meta-analysis (k = 3 studies, total N = 48) showed a large overall improvement in depressive symptoms, *g* = −1.15 [95% CI −2.23, −0.08], *z* = −2.10, *p* = .035. Heterogeneity was high (*Q*(2) = 17.60, *p* <.001; *I*² = 86.2%, τ² = 0.77)), indicating considerable variability across studies. Two studies showed large reductions in depressive symptoms, whereas one study demonstrated only a small change. Sensitivity analyses across alternative assumed pre–post correlations (*r* = 0.3, 0.5, 0.7) yielded similar pooled effects (*g* = -1.00 to -1.34), supporting the robustness of the findings. As above, assessments of funnel plot asymmetry were not performed due to the small number of studies. A forest plot showing standardised mean change (*g*) for pre–post reductions in depression symptoms across studies is shown in **Figure 3**.

As shown, Atmaca et al. (33) and Atmaca et al. (34) had similar very large effect sizes, while Rupp et al. (42) had a small effect size, with a 95% confidence interval that included zero.

Two studies reported Beck Anxiety Inventory (BAI) scores as a secondary outcome. Within-group standardized mean change effect sizes were calculated from the reported pre and post scores. DM was associated with very large reductions in anxiety symptoms in both Atmaca et al. (33) (OCD study, n = 17), *g* = −2.59 [95% CI −3.58, −1.60], and Atmaca et al. (34) (panic disorder study, n = 11), *g* = −1.91 [95% CI −2.90, −0.92]. Given that only two studies reported BAI outcomes, a meta-analysis was not conducted.

### Other results from clinical trials

#### Follow-up

Rupp, Jürgens, et al. (42, OCD patients, n = 20) was the only study to report follow-up data, assessed four weeks post-treatment. Standardised mean change analyses using R indicated that gains achieved with DM were maintained over this period. From pre-treatment to follow-up, a large reduction in obsessive–compulsive symptoms was observed on the Y-BOCS (*g* = −1.74), alongside a small reduction in depressive symptoms (BDI; *g* = −0.31). Examination of post-treatment to follow-up change showed a small additional reduction in OCD symptoms (Y-BOCS; *g* = −0.30) and minimal further reduction in depressive symptoms (BDI; *g* = −0.11).

#### Comparisons

Rupp, Jürgens, et al. (42) compared DM with two other conditions: a Cognitive Restructuring (CR) intervention and a waitlist control. The CR condition matched the DM condition in structure and length (four sessions) but focused on challenging the four cognitive belief domains of the Obsessive Compulsive Cognitions Working Group (OCCWG), excluding the two metacognitive domains (see 67). Standard CBT strategies for cognitive disputation were used.

Both DM and CR produced significantly greater reductions in symptoms than the waitlist control. However, DM and CR did not differ significantly from one another in their effectiveness.

In two additional papers using the same sample Rupp, Falke, et al. (44) and Rupp et al. (43) examined whether DM and CR had effects when measured by ecological momentary assessment (EMA). EMA is a method in which participants report on symptoms, emotions, and behaviours in real time, thereby reducing retrospective bias and increasing ecological validity. EMA was implemented using smartphone prompts randomly delivered ten times per day across four consecutive days, pre and post treatments. Patients were asked to rate several items including the frequency of obsessions as well as associated burden, avoidance, compulsions, DM strategies (e.g., “allowing thoughts to come and go,” “distancing,” “telling oneself it is just a thought”) and CR strategies.

DM and CR produced similar patterns of change. Pre- to post-intervention, participants showed significant reductions in obsession frequency, burden, and avoidance, and increases in the use of both DM and CR strategies. However, there was no evidence that DM uniquely increased the use of DM-specific behaviours relative to CR (or vice versa for CR). There was a trend but no significant reduction in compulsions and no significant changes in emotions.

### Outcomes from the experimental studies

The nine studies that used briefer DM interventions differed in design and outcome measures; therefore, meta-analysis was not considered appropriate. Given this heterogeneity, a narrative approach was used to summarise the findings. Where effect sizes were reported, or were able to be calculated from significant outcomes, they are presented.

#### Symptom-induction studies (seven studies)

Seven studies examined the immediate effects of DM following an induced symptom, trigger, or intrusive thought. Six of these studies reported significant effects of DM on at least one outcome variable, although the size and breadth of these effects varied.

Caselli et al. (36) exposed eight patients with Alcohol Use Disorder to recordings of their alcohol-related thoughts using a counterbalanced design in which each participant underwent both a DM and brief-habituation intervention. They found that DM led to significantly greater reductions in all outcomes: distress, urge to use alcohol, meta-appraisals, and metacognitive beliefs about the alcohol-related thoughts, than the habituation condition. Wilcoxon signed-rank tests were used to analyse changes within each condition. As part of the current review, effect sizes were derived from the reported test statistics, yielding *r-*values ranging from –0.67 to –0.88, indicating large to very large effects.

Gkika and Wells (38) examined the effects of DM and CR on speech-related anxiety in twelve non-clinical participants with elevated social anxiety using a counterbalanced cross-over design. Participants delivered a brief speech and completed baseline measures, then received either DM or CR, delivered a second speech, and were reassessed; the alternative intervention was then administered before a third speech. DM produced significant pre- to post-intervention improvements in all dependent variables: anticipatory processing (*r* = –.63), observer perspective (*r* = –.67), anxiety (*r* = –.64), and negative belief ratings (*r* = –.63), each representing large effects. DM led to significantly larger reductions than CR on all measures but one, with effect sizes for differences being: anticipatory processing (*r* = –.57), observer perspective (*r* = –.45), and negative belief ratings (*r* = –.41), all in the medium-to-large range. Improvements in anxiety did not differ significantly between the two interventions (*r* = –.01). Examination of order effects showed that for one outcome-anticipatory processing-CR led to a significantly worse outcome when given after DM but there was no negative effect when DM was administered after CR.

Wells and Roussis (45) conducted a preliminary study in which 56 non-clinical participants viewed a graphic video depicting road accidents. Participants were then randomly assigned to complete five minutes of DM, acceptance, exposure, or to a control condition in which they were asked to settle down for five minutes before completing the questionnaires. DM was the only condition associated with significantly fewer intrusive images than the control condition, with a Hedges’ *g* of –0.95 (95% CI [–1.73, –0.16]), indicating a large effect.

Ludvik and Boschen (41) exposed 65 non-clinical students to a repeated checking task previously shown to induce OCD-like symptoms involving memory uncertainty and checking behaviour. Participants then completed baseline measures of memory detail, memory confidence, vividness, and outcome confidence. They were subsequently randomly assigned to one of three interventions: DM, CR, or a no-intervention control condition. After the intervention, memory measures were reassessed and participants were allowed to check their responses.

Despite the absence of significant group × time interaction effects, the DM condition showed significant pre to post-intervention improvements in memory detail (*d* = –0.39) and memory confidence (*d* = –0.53). The CR or control condition did not produce significant improvement on these measures. In no condition was there significant change in vividness or outcome confidence.

In terms of checking behaviour, participants in the control condition were significantly more likely to check their responses than those in either the DM group or the CR group. As part of the current review, φ coefficients were calculated from the reported χ² statistics for the pairwise comparisons, yielding φ = .68 and φ = .65, respectively—both representing large effects. There was no significant difference in checking outcomes between the DM and CR conditions.

Akdağ et al. (32) randomly assigned 120 undergraduate students to DM, ATT, slow breathing, or an active control condition. Participants completed a laboratory public-speaking task designed to elicit social anxiety. State metacognition, heart rate variability (HRV), and state anxiety were measured repeatedly from pre-intervention to recovery phases. Additionally, subjective and objective performance of the speech were measured.

There was one significant effect in the DM condition: a pre- to post-intervention HRV increase compared with the control condition. However, no significant findings were reported for DM in other phases or on any other outcome measure. Of the other conditions, only slow breathing produced significant changes—increases in HRV across several phases relative to control, and a reduction in state anxiety immediately post-intervention. Effect sizes for DM relative to control were not reported in a way that permitted conversion to a standardised measure.

Chittaro and Vianello (37) asked 22 students with little meditation experience to identify three personal worries and then practice three thought-distancing techniques: an interactive smartphone app (AEON) in which the written worries were dissolved by simulated waves, a cloud-imagery task (CLOUD), and a card-tossing task (CARD) in which worries were written on cards and thrown away. CLOUD was based on Wells (5) “Cloud Image “ exercise, which is intended to promote detached mindfulness, although—as mentioned earlier—Wells notes that this method involves active engagement with thoughts.

AEON produced significantly higher decentring scores than CARD but not significantly higher than CLOUD, which yielded decentring levels intermediate between the two. CLOUD was rated as less pleasant than AEON and was perceived as more difficult than both AEON and CARD. No effect sizes were reported in a form that permitted conversion to a standardised measure.

Hansmeier et al. (40) examined whether a brief DM intervention could prevent shame following a Thought-Action Fusion (TAF) induction. Eighty-eight participants with elevated (subclinical) OCD symptoms were randomly assigned to a violent TAF induction or a neutral control sentence. The TAF induction significantly increased TAF-state and shame relative to control. Participants in the TAF-induction arm were then randomised to a brief DM intervention (using the cloud metaphor) or a mnemonics control before completing a second TAF induction. DM did not reduce TAF-state or shame, nor did it prevent increases compared with the control condition.

#### DM intervention for pre-existing symptoms (non-clinical; two studies)

Gold and Smout (39) examined the effects of DM on 23 non-clinical participants who experienced high levels of self-critical rumination. Participants identified their typical self-critical rumination triggers and then received a DM session based on Wells’ protocol, followed by daily practice for one week. Significant pre- to post-intervention improvements were reported across several outcomes, and the authors provided *g* values for these changes. Large reductions were observed in self-critical rumination (*g* = 0.94), negative metacognitive beliefs (*g* = 0.93), and inadequate self-criticism (*g* = 1.10), alongside medium-to-large reductions in hated self (*g* = 0.68), positive meta-beliefs (*g* = 0.76), anxiety (*g* = 0.58), and external shame (*g* = 0.66). Large increases were observed in the decentring indices of disidentification (*g* = –1.11) and nonreactivity (*g* = –1.32).

Bolzenkötter et al. (35) examined the short-term effects of DM in daily life using experience sampling methodology. One hundred participants with elevated trait repeated negative thinking (RNT) were randomised to either a DM or an active control condition and completed a 5-day baseline phase followed by a 5-day exercise phase. At each of three daily prompts, participants reported momentary RNT, negative affect, and positive affect before either the DM or active control exercise and again 15 and 30 minutes afterwards. DM exercises used a four-and-a-half-minute audio with guided metaphors (clouds, leaves, trains) instructing participants to observe thoughts as transient without engaging with them. The active control exercises matched the structure, length, and imagery of the DM exercises but excluded all DM-specific elements, instead directing attention to further details of the imagined scenes. Both groups were instructed to find a quiet place and “calm down” before beginning each exercise. Across groups, RNT and affect improved more during the exercise phase than during baseline; however, the DM and active control conditions did not differ at either 15- or 30-minute follow-up. Effects were not reported in a form permitting conversion to *g*.

#### Certainty of Evidence

For clinical trials, certainty of evidence was limited. Only three studies were identified. The adapted Quality Checklist showed that all three studies had deficits in reporting or fulfilling power calculations. Sample sizes were relatively small (11-20) and two of the three studies did not have a control group. There was substantial heterogeneity in results. Additionally, in two studies (33; 34) reporting of missing data was incomplete and it was unclear whether questionnaire items were missing or how missing data were handled.

For experimental studies, certainty of evidence was also limited. All but one study (40) had deficits in power calculations, which were either not reported, incompletely reported, or not fully met. Several studies had smaller samples, namely Caselli et al. (36; n = 8), Gkika and Wells (38; n = 12), and Chittaro and Vianello (37; n = 22). Hansmeier et al. (40) was the only experimental study to fully report missing data and handling procedures. The remaining studies received lower ratings on this criterion on the Quality Checklist. In addition, most experimental studies did not report checks of statistical assumptions.

Overall, the certainty of evidence across both clinical and experimental studies was limited based on number of studies, methodological quality and sample size.

## Discussion

This systematic review aimed to synthesise the evidence on DM delivered as a stand-alone intervention. Fourteen articles were identified, representing twelve independent samples, comprising three clinical trials and nine experimental studies. Although the evidence base remains relatively small, the number and diversity of studies identified indicate a growing area of research and provide an initial basis for evaluating the effects of DM delivered independently. A summary of the main findings of the clinical trials and experimental studies is presented below, followed by discussion of their interpretation, methodological limitations, clinical and theoretical implications, and suggestions for future research.

### Summary of main findings: clinical trials

Two of the clinical trials treated patients with Obsessive–Compulsive Disorder and one patients with Panic Disorder. A meta-analysis of the three clinical trials showed a large pooled effect on primary outcomes, reflecting reductions in OCD and panic symptoms (*g* = −1.80). There was also a large pooled reduction in depressive symptoms, measured as a secondary outcome (*g* = −1.15). Heterogeneity was substantial in both meta-analyses. Inspection of individual study effect sizes suggested that this variability was largely associated with Rupp, Jürgens, et al. (42), which reported smaller effects than the other two trials—although still large for the primary outcome (small for the secondary depressive outcome). Sensitivity analyses indicated that pooled effects were stable across different assumptions regarding pre–post correlations, providing preliminary support for the robustness of these findings. Only two trials (one OCD, one panic disorder) also assessed anxiety as a secondary outcome, which precluded meta-analysis; however, both these trials reported very large pre–post reductions in anxiety.

Follow-up effects were reported in only one study (42), in which gains in the DM condition were maintained and showed some additional improvement at a 4-week follow-up. Rupp, Jürgens, et al. was also the only randomised controlled trial included in the review, and found that DM produced significantly greater symptom reductions than a waitlist control, although it did not differ significantly from the CR condition.

### Summary of main findings: experimental studies

The nine remaining samples employed experimental designs and examined briefer DM interventions, either following symptom or trigger induction or targeting pre-existing symptoms in non-clinical populations. Eight of these nine studies reported significant benefits of DM on at least one outcome, and where effect sizes could be calculated, they were typically in the medium-to-large range. Effects were observed across a diverse set of outcomes, including alcohol-related distress and urges, social anxiety–related processes, metacognitive beliefs, self-critical rumination, negative repetitive thinking, induced intrusions, heart rate variability and checking behaviour. However, the extent of findings across these eight studies was somewhat mixed. While a number of studies reported effects across multiple outcome measures, Akdağ et al. (32) found effects only on a selected outcome from a broader battery of measures. Of note though, participants in that study found DM the hardest intervention to understand and the authors suggest this may have affected their findings. In addition, although Bolzenkötter et al. (35) reported improvements in negative repetitive thinking and accompanying affect, following DM practice, these effects did not significantly exceed those observed in a structurally matched active control condition.

The one study that did not find any significant effects of DM (40) examined outcomes following a Thought–Action Fusion (TAF) induction and found no reduction in TAF or shame following DM. The authors suggested that the brief DM intervention employed may not have been sufficient to counteract the induction.

### Interpretation and possible mechanisms

Findings across both clinical and experimental studies, provide converging, although currently, limited evidence that DM, when delivered on its own, is associated with meaningful change in a range of symptoms and metacognitions.

The overall effect sizes observed for both primary and secondary outcomes in the meta-analyses of the three clinical trials were large. Heterogeneity in effect sizes in the meta-analyses, which appeared to largely reflect the lower effect sizes in Rupp, Jürgens, et al. (42) may partly reflect differences in study design. Rupp, Jürgens, et al. was a randomised controlled trial, whereas the Atmaca studies were open trials. Although the meta-analyses examined within-group change for all studies, randomised controlled trials often produce lower effect sizes due to stricter methodological control and reduced susceptibility to expectancy and other non-specific influences that can inflate outcomes in uncontrolled designs (68, 69). In addition, although all three trials gave four sessions of DM, it was delivered over a shorter period in Rupp, Jürgens, et al. (two weeks) compared to approximately four weeks in the Atmaca studies, which may have allowed less time for symptom improvement.

Despite this heterogeneity, the strong effect sizes in the clinical studies are consistent with the idea that DM may represent a central mechanism of change within MCT, acting on core targets of the treatment—namely the CAS, dysfunctional metacognitive beliefs, and the metacognitive mode (i.e., the manner in which internal experiences are related to).

This interpretation is further supported by evidence from the experimental studies included in the review. Four experimental studies found improvements in CAS-related processes. Of these, three studies found reductions in perseverative thinking (namely rumination, repetitive negative thinking and anticipatory processing) and single studies found improvements in self-focused attention, and checking behaviours. Metacognitive beliefs (including beliefs concerning the usefulness of self-criticism and the uncontrollability or danger of thoughts) decreased in two studies following DM. Improvements in meta-appraisals were found in one of these studies and in one additional study, while shifts toward a detached metacognitive mode, as indexed by measures of decentring, were also found in two studies.

Although further research is needed—particularly on mechanisms that have received little or no attention—such as the impact of DM on sense of self or on attentional and behavioural parts of the CAS-the findings taken together, suggest that DM may operate on core metacognitive processes. Although formal tests of mediation using appropriate longitudinal mediation analyses are needed, results are consistent with the possibility that DM may function as a key active component within MCT.

However, several components of MCT—particularly those aimed at maximally reducing dysfunctional metacognitive beliefs and explicitly addressing relapse prevention—are designed to maintain treatment gains over the longer term. It remains unclear whether DM when delivered alone would be sufficient to sustain change over extended follow-up periods. Only Rupp, Jürgens, et al. (42) examined follow-up and this was only over a four week period. Although gains were maintained, and even further improved over this period, this provides very limited evidence as to the durability of effects. Additional studies with longer follow-up are needed before conclusions can be made as to whether DM alone can achieve sustained change and, if so whether this change is comparable to that found following the full MCT protocol.

### Delivery of DM

There was considerable variation in how DM was delivered across studies, with implications for the interpretation of results. First, procedures varied markedly in length, ranging from a brief instruction in one experimental study to multi-session interventions with between-session homework in the clinical trials. Longer and more structured interventions may allow consolidation of metacognitive principles, thereby producing stronger effects on more trait-like or clinically stable outcomes. Consistent with this, the three clinical trials—each delivering DM over four sessions—demonstrated some of the largest effect sizes and their outcomes were clinical symptoms. Gold and Smout (39) occupy an intermediate position: although their intervention involved a single formal DM session, outcomes included more trait-like processes such as rumination, and the relatively strong effects may reflect the inclusion of several days of subsequent practice. In contrast, brief interventions may primarily influence immediate cognitive or emotional responses rather than more stable psychopathological symptoms. Although strong effects were observed in some experimental studies using brief interventions, these involved short-term outcomes such as induced intrusions or immediate appraisals. Taken together, these findings suggest that intervention length alone is unlikely to account for outcome variability; rather, the interaction between intervention intensity, opportunity for practice, and the state-like versus trait-like nature of the outcome assessed may be important.

A second difference across studies was the techniques used to induce DM, with one study using a brief verbal instruction, others using various metaphors and/or exercises. The extent to which these differing induction techniques influence outcomes remains unclear, and systematic comparison of induction methods is required to determine whether certain formats more effectively induce DM.

Third, in some studies, therapist-guided delivery or brief formulation-based explanations of the rationale for DM may have introduced elements that overlap with aspects of the broader MCT protocol, even though full-protocol MCT was not delivered. Such overlap complicates interpretation of whether effects reflect minimal DM instruction or other aspects of the MCT protocol such as challenging dysfunctional metacognitive beliefs.

Finally, there was variety in modes of delivery, including face-to-face instruction, audio recordings, video materials, and written instructions. These differences may influence participant engagement and/or understanding, thereby contributing to variability in observed outcomes.

Taken together, heterogeneity in intervention length, induction method and delivery mode may all have contributed to differences in outcomes across studies and warrant systematic investigation in future research.

### Fidelity

An issue identified in the review concerned adherence to core DM principles. One study (27) was excluded because, although intended to test DM, it explicitly combined DM with mindfulness instructions involving bodily focus and increased general awareness of thoughts and feelings. Two studies were included despite only partial fidelity to DM principles and therefore received partial scores on the item in the Quality Checklist assessing adherence to Metacognitive Theory. Chittaro and Vianello (37) employed an imagery exercise described by Wells (5) as a method for inducing DM and thus meeting the inclusion criteria of the present review. However, Wells (5) also noted that this imagery-based procedure involved engagement with thoughts and did not represent a pure form of DM, a position clarified further in later developments of the DM construct (1). Bolzenkötter et al. (35) likewise used an imagery-based technique in which the relative contribution of mindfulness and DM elements was unclear.

Neither study reported statistics that permitted calculation of standardised effect sizes, precluding direct comparison with higher-fidelity DM studies. It is possible that results in these studies were influenced by the use of imagery techniques involving conceptual processing, which differ from purer forms of DM. In Bolzenkötter et al. (35), the absence of significant differences relative to a structurally matched active control condition—also involving imagery without DM-specific instructions—may partly reflect the inclusion of imagery within the DM condition itself. Effects might therefore differ when more strictly defined forms of DM are employed. Indeed, the three experimental studies that directly compared purer forms of DM with an active control (36, 38, 40) found superiority of DM on at least some outcome measures. Ensuring theoretical fidelity and clear reporting of DM procedures will be essential for improving interpretability and comparability across future studies.

### Control conditions

Although the primary aim of this review was to examine the initial efficacy of DM, evidence from studies employing control conditions provided important additional information. In the only randomised controlled trial included (42), DM produced significantly greater symptom reduction than a waitlist control but did not differ significantly from CR. This suggests that DM may yield outcomes comparable to an established cognitive intervention. Whether this reflects shared therapeutic mechanisms or distinct but similarly effective mechanisms remains unknown. However, without further randomised controlled trials, including direct head-to-head comparisons with full-protocol MCT, the relative efficacy of DM is still to be established.

As discussed above, the experimental studies with more fidelity to DM principles showed at least some superiority over active controls, while the studies with less fidelity did not. Although the non-superiority findings may be a result of lack of full fidelity, they do raise the possibility that at least some of the effects of DM may come from non-specific factors common to structured psychological tasks—such as expectancy effects, attentional engagement, cognitive activation, or general stress reduction—rather than mechanisms unique to DM. At the same time, the superiority of DM over some active comparisons suggests that specific metacognitive processes may contribute meaningfully to observed effects. The results from two studies finding superiority over CR raise the possibility of a key difference, at least with briefer interventions, between cognitive engagement with thoughts to modify their content and DM processes of detachment and non-engagement with thoughts. Future studies should include structurally equivalent active controls as well as process measures capable of distinguishing between changes attributable to core DM processes and those arising from features shared across intervention conditions. As one study reported a potential order effect, with reduced effects on one measure when CR followed DM, future research should consider intervention sequencing when combining treatments as this may have clinical implications.

### Limitations

The findings of this review should be interpreted in light of several important limitations.

First, the overall evidence base for DM delivered as a stand-alone intervention is currently small. Only three clinical trials were identified, each with modest sample sizes and limited to two diagnostic groups (OCD and panic disorder). This constrains the strength of conclusions and limits generalisability. In addition, the meta-analysis was confined to post-treatment outcomes, as follow-up data were reported in only one study, which was only over a short time frame. Consequently, the durability of effects and the risk of relapse following DM delivered alone remain largely unknown. Accordingly, conclusions regarding clinical efficacy should be regarded as initial and provisional rather than definitive.

Although nine experimental studies were identified, sample sizes were often small and the substantial variability across these studies meant that no individual symptom domain was examined in large numbers. Designs varied widely (e.g., cross-over, pre–post, and between-group experiments), as did target processes. This heterogeneity precluded meta-analysis and necessitated narrative synthesis. While this diversity of outcomes measured provides initial indications of potential transdiagnostic applicability, it limits conclusions as to the effects of DM on any specific symptom or mechanism.

Second, concerns relating to risk of bias and reporting quality were identified across studies. Assessment using the adapted Quality Checklist revealed recurrent limitations, including insufficient justification of sample sizes or power calculations, incomplete reporting of missing data and attrition, and variable transparency in statistical methods (e.g., reporting of assumptions, handling of outliers, and adjustment for multiple testing). Several studies did not report outcomes in a format that permitted calculation of standardised effect sizes, limiting comparability across studies. Collectively, these methodological limitations reduce certainty in the findings.

Third, the review protocol for the current study was not pre-registered. While eligibility criteria and key methodological procedures were determined in advance, formal pre-registration would have strengthened transparency.

Fourth, to ensure methodological quality and allow detailed study appraisal, grey literature sources (e.g., conference abstracts, dissertations, and preprints), trial registries, and non-English language publications were not systematically searched. However, this approach may have resulted in the omission of relevant studies and introduces the potential for publication and language bias.

Fifth, heterogeneity in DM implementation introduces additional uncertainty. Interventions varied in duration, induction method, fidelity to metacognitive theory, delivery format, and degree of overlap with broader MCT components. Such variability makes it difficult to isolate the effects attributable specifically to core DM processes and may contribute to variability in outcomes across studies.

Finally, generalisability is limited by sample characteristics. All but one of the experimental studies used non-clinical samples, most often undergraduate students. It remains unclear whether findings from these experimental paradigms generalise to clinical populations characterised by higher symptom severity or chronicity. On the one hand, individuals with elevated symptoms may show greater scope for improvement; on the other, entrenched patterns of responding or reduced attentional flexibility could attenuate effects. Gender imbalance—reflected in the predominance of female participants in the three clinical trials and in most of the experimental studies—further limits interpretation, as potential differences in metacognitive beliefs or self-regulatory styles across genders may influence responsiveness to DM. No studies examined children despite emerging evidence for the efficacy of full-protocol MCT in younger populations and older adults were not specifically investigated.

### Suggestions for future research

The present review highlights several important directions for future research.

First, adequately powered randomised controlled trials are needed comparing DM delivered alone with structurally equivalent active control conditions, as well as with full-protocol MCT. Such head-to-head designs would help clarify the relative efficacy of DM and help determine whether observed effects reflect core metacognitive mechanisms or broader non-specific therapeutic factors.

Second, longer-term follow-up is essential as only one study included short-term follow-up data. Future trials, and experimental studies where appropriate, should incorporate follow-up. For clinical trials, these should include multiple assessment points over extended periods to examine maintenance of gains and whether this is linked to ongoing practice.

Third, systematic evaluation of induction methods and delivery formats is warranted. Studies should directly compare brief single-session inductions with multi-session protocols, compare different ways of inducing DM, as well as different modes of delivery (e.g., therapist-led, audio, written, or video formats). In doing so it will be important to maintain conceptual and procedural integrity in the choice and implementation of “true” DM techniques. According to metacognitive theory DM should not involve elements that transform or challenge thoughts, have the goal of removing them or introduce more thinking. Reporting of how fidelity to metacognitive theory principles is ensured should be included in future research. Additionally, inclusion of mechanism-focused measures—such as changes in CAS processes, metacognitive beliefs, and metacognitive mode—will be critical for testing theoretical predictions and identifying active components.

Finally, research should prioritise underrepresented populations. Clinical trials are needed across a broader range of diagnostic groups beyond OCD and panic disorder. Clinical and experimental studies are particularly needed in children, adolescents, older adults, more gender-balanced samples, and culturally diverse populations to examine the generalisability of findings.

## Conclusion

This systematic review and meta-analysis provides early-stage evidence that DM, when delivered as a stand-alone intervention, can produce large symptom reductions in clinical trials, and meaningful changes in experimentally induced or pre-existing symptoms. Findings were also consistent with theoretical predictions that DM may modify core metacognitive processes implicated in psychological difficulties. At the same time, the current evidence base has significant limitations and substantial further research is required to examine the validity, durability and generalisability of current findings.

With further research, it may become clearer whether DM is a central therapeutic component within MCT, as well as potentially an intervention in its own right. Meanwhile, current findings dovetail with the growing literature on ATT, which has also demonstrated large effects when delivered as a stand-alone intervention. Taken together, the emerging DM and ATT literatures point towards the potential of brief, focused metacognitive interventions as effective and efficient methods to alleviate psychological distress.

## Data Availability

The original contributions presented in the study are included in the article/**Supplementary Material**. Further inquiries can be directed to the corresponding author.
