# Supplementary material for: Detached mindfulness as a stand-alone intervention: a Systematic Review and meta-analysis
Source: Front Psychiatry. 2026 Apr 15;17:1771705. doi: 10.3389/fpsyt.2026.1771705 (PMC13125881; doi:10.3389/fpsyt.2026.1771705)
Supplement: Supplementary file 1 [file Table1.docx]

############################################################

# Detached Mindfulness – Meta-Analyses (RGui interactive)

# Shows ONLY TWO forest plots (Option A): Primary + BDI

# Primary: Y-BOCS / Panic severity (3 studies)

# Secondary: BDI (3 studies)

# Sensitivity checks for assumed pre–post r (0.3/0.5/0.7)

############################################################

# ---- Packages ----

# install.packages("metafor") # run once if needed

library(metafor)

# ---- Helper: close all open graphics devices (Windows/RGui) ----

close_all_devices <- function() {

while (dev.cur() > 1) dev.off()

}

# Start clean (optional)

close_all_devices()

############################################################

# 1) PRIMARY OUTCOME: anxiety-related symptoms (Y-BOCS / Panic)

############################################################

primary_labels <- c(

"Atmaca et al., 2024a",

"Rupp, Jürgens, et al., 2019",

"Atmaca et al., 2024b"

)

primary_data <- data.frame(

study = primary_labels,

disorder = c("OCD", "OCD", "Panic"),

n = c(17, 20, 11),

mean_pre = c(25.29, 24.30, 17.36),

sd_pre = c(5.70, 4.00, 2.58),

mean_post = c(13.53, 19.05, 9.64),

sd_post = c(3.41, 6.30, 3.01),

r_prepost = c(0.5, 0.5, 0.5) # assumed if not reported

)

primary_es <- escalc(

measure = "SMCC",

m1i = mean_post,

m2i = mean_pre,

sd1i = sd_post,

sd2i = sd_pre,

ni = n,

ri = r_prepost,

data = primary_data

)

cat("\n================ PRIMARY (Y-BOCS / Panic) ================\n")

print(primary_es[, c("study", "yi", "vi")])

# Random-effects model (REML)

res_primary <- rma(yi, vi, data = primary_es, method = "REML")

cat("\n--- Random-effects (REML) model summary ---\n")

print(summary(res_primary))

# Sensitivity to assumed pre–post correlation r

cat("\n--- Sensitivity to assumed pre–post correlation r ---\n")

for (r in c(0.3, 0.5, 0.7)) {

es_r <- escalc(

"SMCC",

m1i = primary_data$mean_post, m2i = primary_data$mean_pre,

sd1i = primary_data$sd_post, sd2i = primary_data$sd_pre,

ni = primary_data$n, ri = r,

data = primary_data

)

fit_r <- rma(yi, vi, data = es_r, method = "REML")

cat(

"PRIMARY: assumed r=", r,

" -> pooled g=", round(as.numeric(fit_r$b), 3),

" [", round(fit_r$ci.lb, 3), ", ", round(fit_r$ci.ub, 3), "]",

" ; I^2=", round(fit_r$I2, 1), "%\n",

sep = ""

)

}

############################################################

# 2) SECONDARY OUTCOME: depressive symptoms (BDI)

############################################################

bdi_data <- data.frame(

study = primary_labels,

disorder = c("OCD", "OCD", "Panic"),

n = c(17, 20, 11),

mean_pre = c(24.24, 16.55, 21.82),

sd_pre = c(7.51, 10.66, 6.69),

mean_post = c(12.41, 14.55, 10.91),

sd_post = c(3.76, 12.71, 2.88),

r_prepost = c(0.5, 0.5, 0.5) # assumed if not reported

)

bdi_es <- escalc(

measure = "SMCC",

m1i = mean_post,

m2i = mean_pre,

sd1i = sd_post,

sd2i = sd_pre,

ni = n,

ri = r_prepost,

data = bdi_data

)

cat("\n================ SECONDARY (BDI) ================\n")

print(bdi_es[, c("study", "yi", "vi")])

res_bdi <- rma(yi, vi, data = bdi_es, method = "REML")

cat("\n--- Random-effects (REML) model summary ---\n")

print(summary(res_bdi))

# Sensitivity to assumed pre–post correlation r

cat("\n--- Sensitivity to assumed pre–post correlation r ---\n")

for (r in c(0.3, 0.5, 0.7)) {

es_r <- escalc(

"SMCC",

m1i = bdi_data$mean_post, m2i = bdi_data$mean_pre,

sd1i = bdi_data$sd_post, sd2i = bdi_data$sd_pre,

ni = bdi_data$n, ri = r,

data = bdi_data

)

fit_r <- rma(yi, vi, data = es_r, method = "REML")

cat(

"BDI: assumed r=", r,

" -> pooled g=", round(as.numeric(fit_r$b), 3),

" [", round(fit_r$ci.lb, 3), ", ", round(fit_r$ci.ub, 3), "]",

" ; I^2=", round(fit_r$I2, 1), "%\n",

sep = ""

)

}

############################################################

# 3) FOREST PLOTS (Option A): TWO SEPARATE RGui WINDOWS

# (No other forest plots are produced.)

############################################################

# --- Primary forest plot window ---

windows(width = 12, height = 9)

forest(

res_primary,

slab = primary_data$study,

xlab = "Standardized Mean Change (Hedges' g; primary anxiety outcome)",

cex = 1.8,

cex.axis = 1.6,

cex.lab = 1.8,

pch = 15,

psize = 2

)

abline(v = 0, lty = 2)

# --- BDI forest plot window ---

windows(width = 12, height = 9)

forest(

res_bdi,

slab = bdi_data$study,

xlab = "Standardized Mean Change (Hedges' g; BDI pre–post)",

cex = 1.8,

cex.axis = 1.6,

cex.lab = 1.8,

pch = 15,

psize = 2

)

abline(v = 0, lty = 2)

############################################################

# Note:

# With k < 10, do not run/interpret funnel plots or Egger tests.

############################################################

############################################################

# Rupp et al. (2019)

# Detached Mindfulness group

# Pre–Follow-up and Post–Follow-up effect sizes

# Using SMCC (Hedges g) with assumed r = .5

############################################################

library(metafor)

############################################################

# Y-BOCS

############################################################

ybocs_fu <- data.frame(

time = c("Pre–Follow-up", "Post–Follow-up"),

mean1 = c(17.55, 17.55), # follow-up mean

mean2 = c(24.30, 19.05), # pre and post means

sd1 = c(6.03, 6.03), # follow-up SD

sd2 = c(4.00, 6.30), # pre and post SD

n = c(20,20),

r = c(.5,.5)

)

ybocs_es <- escalc(

measure="SMCC",

m1i=mean1,

m2i=mean2,

sd1i=sd1,

sd2i=sd2,

ni=n,

ri=r,

data=ybocs_fu

)

ybocs_es$sei <- sqrt(ybocs_es$vi)

ybocs_es$ci_lb <- ybocs_es$yi - 1.96*ybocs_es$sei

ybocs_es$ci_ub <- ybocs_es$yi + 1.96*ybocs_es$sei

ybocs_es

############################################################

# BDI

############################################################

bdi_fu <- data.frame(

time = c("Pre–Follow-up", "Post–Follow-up"),

mean1 = c(13.30, 13.30),

mean2 = c(16.55, 14.55),

sd1 = c(11.23,11.23),

sd2 = c(10.66,12.71),

n=c(20,20),

r=c(.5,.5)

)

bdi_es <- escalc(

measure="SMCC",

m1i=mean1,

m2i=mean2,

sd1i=sd1,

sd2i=sd2,

ni=n,

ri=r,

data=bdi_fu

)

bdi_es$sei <- sqrt(bdi_es$vi)

bdi_es$ci_lb <- bdi_es$yi - 1.96*bdi_es$sei

bdi_es$ci_ub <- bdi_es$yi + 1.96*bdi_es$sei

bdi_es

############################################################

# Print clean output

############################################################

cat("\nY-BOCS effects\n")

print(ybocs_es[,c("time","yi","ci_lb","ci_ub")])

cat("\nBDI effects\n")

print(bdi_es[,c("time","yi","ci_lb","ci_ub")])
