# Supplementary material for: Detached mindfulness as a stand-alone intervention: a Systematic Review and meta-analysis
Source: Front Psychiatry. 2026 Apr 15;17:1771705. doi: 10.3389/fpsyt.2026.1771705 (PMC13125881; doi:10.3389/fpsyt.2026.1771705)
Supplement: Supplementary file 2 [file Table2.docx]

############################################################

# Effect size calculations for individual experimental studies

# Wilcoxon signed-rank tests and chi-square tests

############################################################

############################################################

# Caselli et al. (AUD experiment)

# Wilcoxon signed-rank test: z -> effect size r

#

# Formula:

# r = z / sqrt(N)

#

# where:

# z = Wilcoxon test statistic

# N = number of paired observations

############################################################

# Sample size

N <- 8

# Reported Wilcoxon z values

caselli <- data.frame(

outcome = c(

"Fear of alcohol-related thoughts",

"Perceived uncontrollability",

"Need to control drinking thoughts",

"Thought–action fusion belief",

"Distress",

"Urge to drink"

),

z = c(-2.5, -1.9, -2.4, -2.0, -2.0, -2.3)

)

# Compute effect size r

caselli$r <- caselli$z / sqrt(N)

# Absolute value (for interpretation)

caselli$abs_r <- abs(caselli$r)

# Magnitude labels (common guidelines)

caselli$magnitude <- cut(

caselli$abs_r,

breaks = c(-Inf, .10, .30, .50, Inf),

labels = c("trivial", "small", "medium", "large"),

right = FALSE

)

cat("\nCaselli et al. Wilcoxon effect sizes:\n")

print(

data.frame(

Outcome = caselli$outcome,

z = caselli$z,

r = round(caselli$r, 3),

Magnitude = caselli$magnitude

),

row.names = FALSE

)

############################################################

# Ludvik & Boschen (2015)

# Chi-square effect sizes

#

# Overall test: Cramer's V

# Pairwise tests: phi

############################################################

# Sample sizes

n_DM <- 21

n_CR <- 22

n_Control <- 22

N_total <- n_DM + n_CR + n_Control

# ---- Function: Cramer's V ----

cramers_v <- function(chi2, N, r, c)

{

k <- min(r - 1, c - 1)

sqrt(chi2 / (N * k))

}

# ---- Function: phi ----

phi <- function(chi2, N)

{

sqrt(chi2 / N)

}

# Overall chi-square

chi2_overall <- 24.95

V <- cramers_v(

chi2_overall,

N_total,

r = 3,

c = 2

)

cat("\nLudvik & Boschen overall effect size:\n")

cat(

"Cramer's V =",

round(V, 3),

"\n"

)

# Pairwise comparisons

pairwise <- data.frame(

Comparison = c(

"DM vs Control",

"CR vs Control",

"DM vs CR"

),

chi2 = c(

19.88,

18.43,

0.068

),

N = c(

n_DM + n_Control,

n_CR + n_Control,

n_DM + n_CR

)

)

pairwise$phi <- phi(

pairwise$chi2,

pairwise$N

)

cat("\nLudvik & Boschen pairwise effect sizes:\n")

print(

data.frame(

Comparison = pairwise$Comparison,

phi = round(pairwise$phi, 3)

),

row.names = FALSE

)

############################################################

# End of script

############################################################
